# Methyl (2*E*)-2-{[(2-methyl­quinolin-8-yl)­oxy]meth­yl}-3-(thio­phen-2-yl)acrylate

**DOI:** 10.1107/S1600536812014560

**Published:** 2012-04-21

**Authors:** S. Anand, S. Narayanan, S. Sundaramoorthy, D. Velmurugan

**Affiliations:** a Postgraduate and Research Department of Chemistry, Presidency College, Chennai 600 005, India; b Centre of Advanced Study in Crystallography and Biophysics, University of Madras, Guindy Campus, Chennai 600 025, India

## Abstract

In the mol­ecule of the title compound, C_19_H_17_NO_3_S, the dihedral angle formed by the quinoline ring system and the thio­phene ring is 83.15 (8)°. In the crystal, C—H⋯O hydrogen bonds link the mol­ecules into a *C*(8) chain running along the *b* axis. The packing of the mol­ecules is further influenced by C—H⋯π inter­actions.

## Related literature

For the biological activity of thienyl acrylate and thio­phene derivatives, see: Anand *et al.* (2011 ▶); Ferreira *et al.* (2006 ▶); Bonini *et al.* (2005 ▶). For general background to quinoline derivatives, see: Mali *et al.* (2010 ▶). For a related structure, see: Prasath *et al.* (2011 ▶).
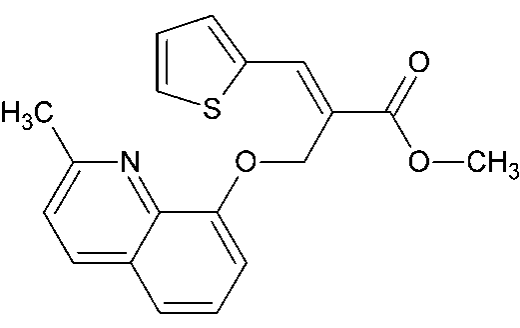


## Experimental

### 

#### Crystal data

C_19_H_17_NO_3_S
*M* *_r_* = 339.40
Orthorhombic, 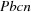

*a* = 24.545 (8) Å
*b* = 8.689 (3) Å
*c* = 15.809 (5) Å
*V* = 3371.5 (19) Å^3^
*Z* = 8
Mo *K*α radiation
μ = 0.21 mm^−1^
*T* = 293 K
0.25 × 0.23 × 0.2 mm

#### Data collection

Bruker SMART APEXII area-detector diffractometer
Absorption correction: multi-scan (*SADABS*; Bruker, 2008 ▶) *T*_min_ = 0.949, *T*_max_ = 0.959
17529 measured reflections
4152 independent reflections
2805 reflections with *I* > 2σ(*I*)
*R*_int_ = 0.032

#### Refinement

*R*[*F* ^2^ > 2σ(*F* ^2^)] = 0.041
*wR*(*F* ^2^) = 0.122
*S* = 1.03
4152 reflections
219 parameters
H-atom parameters constrained
Δρ_max_ = 0.23 e Å^−3^
Δρ_min_ = −0.21 e Å^−3^

### 

Data collection: *APEX2* (Bruker, 2008 ▶); cell refinement: *SAINT* (Bruker, 2008 ▶); data reduction: *SAINT*; program(s) used to solve structure: *SHELXS97* (Sheldrick, 2008 ▶); program(s) used to refine structure: *SHELXL97* (Sheldrick, 2008 ▶); molecular graphics: *ORTEP-3* (Farrugia, 1997 ▶); software used to prepare material for publication: *SHELXL97* and *PLATON* (Spek, 2009 ▶).

## Supplementary Material

Crystal structure: contains datablock(s) global, I. DOI: 10.1107/S1600536812014560/bt5861sup1.cif

Structure factors: contains datablock(s) I. DOI: 10.1107/S1600536812014560/bt5861Isup2.hkl

Supplementary material file. DOI: 10.1107/S1600536812014560/bt5861Isup3.cml

Additional supplementary materials:  crystallographic information; 3D view; checkCIF report

## Figures and Tables

**Table 1:** Hydrogen-bond geometry (Å, °) *Cg*3 is the centroid of the C1/C2/C7–C10 ring.

*D*—H⋯*A*	*D*—H	H⋯*A*	*D*⋯*A*	*D*—H⋯*A*
C19—H19*B*⋯*Cg*3^i^	0.96	2.89	3.505 (2)	123
C17—H17⋯O3^ii^	0.93	2.48	3.056 (2)	120

Symmetry codes: (i) 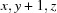; (ii) 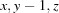.

**Fig. 1.**
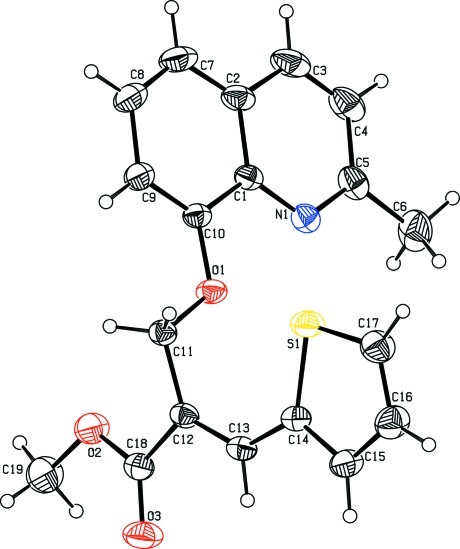
A perspective view of the molecule showing the thermal ellipsoids drawn at the 30% probability level.

**Fig. 2.**
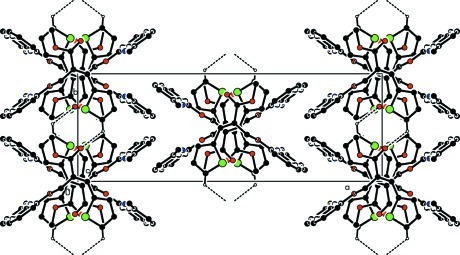
C—H···O interactions (dotted lines) in the crystal structure of the title compound. The crystal packing of the molecules is viewed down the c axis.

## supplementary crystallographic information

### Comment

The title compound similar to the derivatives reported is found to exhibit remarkable antibacterial activity (Anand *et al.*, 2011). Thiophene containing compounds are well known to exhibit various biological activities such as antioxidant activity (Ferreira *et al.*, 2006), anti-inflammatory agents and anti-HIV PR inhibitors (Bonini *et al.*, 2005). Quinolines have gained importance in medicinal and natural product chemistry due to their interesting biological and pharmacological activities. They possess anti-malarial, anti-tuberculosis, anti-inflammatory and anti-cancer properties (Mali *et al.*, 2010). In order to get detailed information such as the geometrical features and the underlying interaction of the crystal structure, an X-ray study of the title compound was carried out.

In the title compound (Fig. 1), the quinoline ring system is essentially planar with a maximum deviation of 0.007 (2) Å at atom C5. This mean plane of the quinoline ring forms a dihedral angle of 83.15 (8)° with the thiophene ring. The methyl acrylate group assumes an extended conformation as can be seen from torsion angles C12—C18—O2—C19 [-179.2 (1)°] and C13—C12—C18—O2 [-169.6 (1)°].

The atom C15 in the molecule (*x*,*y*,*z*) donates one proton to atom O4 of the molecule at (*x*,-1 + *y*,*z*) forming a C(8) chain running along the *b* axis (Fig. 2). The packing of the molecules is further influenced by C—H···π interactions.

### Experimental

Methyl (2*Z*)-2-(bromo methyl)-3-(2-thienyl) acrylate (0.006 mole) was treated with 2-methyl-8-hydroxy quinoline (0.006 mole) in the presence of potassium carbonate (0.006 mole) in dry dimethylformamide 10 ml for 1hr at room temperature. Water was added to the reaction mixture and stirred for 30 min. Thus the obtained crude thienyl acrylate was separated by filtration and washed with methanol. Recrystalization of the compound from ethanol gave X-ray diffraction quality crystals of methyl (2E)-2-{[(2-methylquinolin-8-yl) oxy] methyl}-3-(2-thienyl) acrylate.

### Refinement

H atoms were positioned geometrically (C—H = 0.93–0.98 Å) and allowed to ride on their parent atoms, with 1.5*U*_eq_(C) for methyl H and 1.2 *U*_eq_(C) for other H atoms.

### Crystal data

**Table:**

C_19_H_17_NO_3_S	*F*(000) = 1424
*M**_r_* = 339.40	*D*_x_ = 1.337 Mg m^−^^3^
Orthorhombic, *P**b**c**n*	Mo *K*α radiation, λ = 0.71073 Å
Hall symbol: -P 2n 2ab	Cell parameters from 2025 reflections
*a* = 24.545 (8) Å	θ = 1.7–28.3°
*b* = 8.689 (3) Å	µ = 0.21 mm^−^^1^
*c* = 15.809 (5) Å	*T* = 293 K
*V* = 3371.5 (19) Å^3^	Block, colourless
*Z* = 8	0.25 × 0.23 × 0.2 mm

### Data collection

**Table:**

Bruker SMART APEXII area-detector diffractometer	4152 independent reflections
Radiation source: fine-focus sealed tube	2805 reflections with *I* > 2σ(*I*)
Graphite monochromator	*R*_int_ = 0.032
ω and φ scans	θ_max_ = 28.3°, θ_min_ = 1.7°
Absorption correction: multi-scan (*SADABS*; Bruker, 2008)	*h* = −32→32
*T*_min_ = 0.949, *T*_max_ = 0.959	*k* = −11→9
17529 measured reflections	*l* = −20→20

### Refinement

**Table:**

Refinement on *F*^2^	Primary atom site location: structure-invariant direct methods
Least-squares matrix: full	Secondary atom site location: difference Fourier map
*R*[*F*^2^ > 2σ(*F*^2^)] = 0.041	Hydrogen site location: inferred from neighbouring sites
*wR*(*F*^2^) = 0.122	H-atom parameters constrained
*S* = 1.03	*w* = 1/[σ^2^(*F*_o_^2^) + (0.0611*P*)^2^ + 0.3929*P*] where *P* = (*F*_o_^2^ + 2*F*_c_^2^)/3
4152 reflections	(Δ/σ)_max_ < 0.001
219 parameters	Δρ_max_ = 0.23 e Å^−^^3^
0 restraints	Δρ_min_ = −0.21 e Å^−^^3^

### Special details

**Table:**

Geometry. All e.s.d.'s (except the e.s.d. in the dihedral angle between two l.s. planes) are estimated using the full covariance matrix. The cell e.s.d.'s are taken into account individually in the estimation of e.s.d.'s in distances, angles and torsion angles; correlations between e.s.d.'s in cell parameters are only used when they are defined by crystal symmetry. An approximate (isotropic) treatment of cell e.s.d.'s is used for estimating e.s.d.'s involving l.s. planes.
Refinement. Refinement of *F*^2^ against ALL reflections. The weighted *R*-factor *wR* and goodness of fit *S* are based on *F*^2^, conventional *R*-factors *R* are based on *F*, with *F* set to zero for negative *F*^2^. The threshold expression of *F*^2^ > σ(*F*^2^) is used only for calculating *R*-factors(gt) *etc*. and is not relevant to the choice of reflections for refinement. *R*-factors based on *F*^2^ are statistically about twice as large as those based on *F*, and *R*- factors based on ALL data will be even larger.

### Fractional atomic coordinates and isotropic or equivalent isotropic displacement parameters (Å^2^)

**Table:**

	*x*	*y*	*z*	*U*_iso_*/*U*_eq_	
C1	0.66864 (5)	0.24488 (16)	0.06658 (11)	0.0385 (4)	
C2	0.71433 (6)	0.15068 (18)	0.08567 (13)	0.0498 (5)	
C3	0.74634 (7)	0.1053 (2)	0.01550 (16)	0.0656 (6)	
H3	0.7770	0.0443	0.0240	0.079*	
C4	0.73280 (7)	0.1497 (2)	−0.06405 (16)	0.0643 (6)	
H4	0.7541	0.1193	−0.1098	0.077*	
C5	0.68630 (6)	0.2421 (2)	−0.07710 (12)	0.0516 (4)	
C6	0.66978 (9)	0.2936 (3)	−0.16408 (14)	0.0752 (6)	
H6A	0.6315	0.2762	−0.1717	0.113*	
H6B	0.6898	0.2362	−0.2056	0.113*	
H6C	0.6775	0.4013	−0.1705	0.113*	
C7	0.72541 (7)	0.1091 (2)	0.17010 (15)	0.0628 (5)	
H7	0.7554	0.0475	0.1824	0.075*	
C8	0.69243 (7)	0.1587 (2)	0.23369 (14)	0.0579 (5)	
H8	0.7001	0.1303	0.2891	0.070*	
C9	0.64683 (6)	0.25249 (19)	0.21687 (11)	0.0471 (4)	
H9	0.6248	0.2858	0.2611	0.056*	
C10	0.63489 (6)	0.29474 (17)	0.13523 (11)	0.0378 (4)	
C11	0.55580 (6)	0.43432 (17)	0.17728 (10)	0.0385 (3)	
H11A	0.5761	0.4878	0.2210	0.046*	
H11B	0.5378	0.3464	0.2027	0.046*	
C12	0.51445 (6)	0.54034 (17)	0.13842 (10)	0.0383 (3)	
C13	0.46895 (6)	0.49681 (18)	0.09823 (10)	0.0396 (4)	
H13	0.4484	0.5781	0.0771	0.048*	
C14	0.44592 (6)	0.34661 (17)	0.08167 (11)	0.0415 (4)	
C15	0.39555 (7)	0.32582 (19)	0.04619 (13)	0.0517 (4)	
H15	0.3733	0.4066	0.0290	0.062*	
C16	0.38082 (8)	0.1701 (2)	0.03840 (15)	0.0622 (5)	
H16	0.3477	0.1372	0.0162	0.075*	
C17	0.41989 (7)	0.0735 (2)	0.06666 (13)	0.0604 (5)	
H17	0.4169	−0.0332	0.0660	0.072*	
C18	0.52379 (6)	0.70908 (19)	0.14450 (12)	0.0453 (4)	
C19	0.58520 (9)	0.9061 (2)	0.18099 (16)	0.0772 (7)	
H19A	0.5752	0.9586	0.1299	0.116*	
H19B	0.6234	0.9199	0.1914	0.116*	
H19C	0.5648	0.9475	0.2275	0.116*	
N1	0.65531 (5)	0.28865 (15)	−0.01364 (9)	0.0434 (3)	
O1	0.59204 (4)	0.38425 (13)	0.11118 (7)	0.0437 (3)	
O2	0.57341 (5)	0.74353 (13)	0.17233 (9)	0.0614 (4)	
O3	0.49055 (6)	0.80581 (14)	0.12741 (11)	0.0718 (5)	
S1	0.475215 (18)	0.16968 (5)	0.10362 (3)	0.05398 (16)	

### Atomic displacement parameters (Å^2^)

**Table:**

	*U*^11^	*U*^22^	*U*^33^	*U*^12^	*U*^13^	*U*^23^
C1	0.0292 (6)	0.0331 (7)	0.0532 (10)	−0.0013 (5)	−0.0009 (6)	−0.0032 (7)
C2	0.0326 (7)	0.0414 (8)	0.0753 (14)	0.0045 (6)	−0.0021 (8)	−0.0043 (9)
C3	0.0393 (9)	0.0571 (11)	0.1005 (18)	0.0135 (8)	0.0081 (10)	−0.0103 (12)
C4	0.0466 (10)	0.0657 (12)	0.0806 (16)	0.0037 (8)	0.0205 (10)	−0.0188 (12)
C5	0.0426 (8)	0.0529 (10)	0.0594 (12)	−0.0063 (7)	0.0110 (8)	−0.0130 (9)
C6	0.0673 (13)	0.1046 (17)	0.0536 (13)	0.0006 (12)	0.0134 (10)	−0.0128 (13)
C7	0.0435 (9)	0.0585 (10)	0.0864 (16)	0.0137 (8)	−0.0141 (10)	0.0079 (11)
C8	0.0513 (10)	0.0604 (11)	0.0621 (13)	0.0059 (8)	−0.0169 (9)	0.0089 (10)
C9	0.0440 (8)	0.0479 (9)	0.0493 (11)	0.0032 (7)	−0.0048 (7)	0.0027 (9)
C10	0.0319 (7)	0.0341 (7)	0.0475 (10)	0.0012 (5)	−0.0021 (6)	0.0019 (7)
C11	0.0370 (7)	0.0404 (8)	0.0380 (9)	0.0048 (6)	0.0042 (6)	0.0013 (7)
C12	0.0371 (8)	0.0386 (7)	0.0392 (9)	0.0065 (6)	0.0076 (6)	0.0014 (7)
C13	0.0375 (7)	0.0377 (7)	0.0437 (10)	0.0074 (6)	0.0064 (7)	0.0049 (7)
C14	0.0401 (8)	0.0394 (8)	0.0448 (10)	0.0059 (6)	0.0013 (7)	0.0045 (7)
C15	0.0451 (9)	0.0466 (9)	0.0634 (13)	0.0031 (7)	−0.0078 (8)	0.0060 (9)
C16	0.0514 (10)	0.0573 (11)	0.0778 (15)	−0.0074 (8)	−0.0133 (10)	0.0012 (10)
C17	0.0643 (11)	0.0429 (9)	0.0740 (14)	−0.0046 (8)	−0.0061 (10)	0.0021 (10)
C18	0.0462 (9)	0.0416 (8)	0.0479 (11)	0.0053 (7)	0.0016 (7)	0.0007 (8)
C19	0.0833 (15)	0.0476 (11)	0.1007 (19)	−0.0120 (9)	−0.0250 (13)	−0.0013 (12)
N1	0.0353 (6)	0.0454 (7)	0.0496 (9)	−0.0011 (5)	0.0047 (6)	−0.0049 (7)
O1	0.0397 (6)	0.0511 (6)	0.0403 (7)	0.0156 (5)	0.0053 (4)	0.0047 (5)
O2	0.0562 (7)	0.0449 (7)	0.0830 (10)	−0.0036 (5)	−0.0169 (6)	−0.0003 (7)
O3	0.0635 (8)	0.0401 (6)	0.1119 (14)	0.0101 (6)	−0.0197 (8)	−0.0007 (7)
S1	0.0524 (3)	0.0389 (2)	0.0707 (4)	0.00640 (17)	−0.0103 (2)	0.0031 (2)

### Geometric parameters (Å, º)

**Table:**

C1—N1	1.364 (2)	C11—C12	1.502 (2)
C1—C2	1.421 (2)	C11—H11A	0.9700
C1—C10	1.432 (2)	C11—H11B	0.9700
C2—C7	1.409 (3)	C12—C13	1.339 (2)
C2—C3	1.416 (3)	C12—C18	1.487 (2)
C3—C4	1.357 (3)	C13—C14	1.446 (2)
C3—H3	0.9300	C13—H13	0.9300
C4—C5	1.411 (3)	C14—C15	1.370 (2)
C4—H4	0.9300	C14—S1	1.7323 (16)
C5—N1	1.322 (2)	C15—C16	1.406 (2)
C5—C6	1.502 (3)	C15—H15	0.9300
C6—H6A	0.9600	C16—C17	1.351 (3)
C6—H6B	0.9600	C16—H16	0.9300
C6—H6C	0.9600	C17—S1	1.6983 (19)
C7—C8	1.361 (3)	C17—H17	0.9300
C7—H7	0.9300	C18—O3	1.2021 (19)
C8—C9	1.410 (2)	C18—O2	1.3292 (19)
C8—H8	0.9300	C19—O2	1.448 (2)
C9—C10	1.373 (2)	C19—H19A	0.9600
C9—H9	0.9300	C19—H19B	0.9600
C10—O1	1.3622 (17)	C19—H19C	0.9600
C11—O1	1.4396 (17)		
			
N1—C1—C2	123.20 (15)	C12—C11—H11A	110.1
N1—C1—C10	118.78 (13)	O1—C11—H11B	110.1
C2—C1—C10	118.01 (16)	C12—C11—H11B	110.1
C7—C2—C3	124.31 (17)	H11A—C11—H11B	108.5
C7—C2—C1	120.08 (17)	C13—C12—C18	115.95 (14)
C3—C2—C1	115.61 (18)	C13—C12—C11	125.75 (14)
C4—C3—C2	120.75 (17)	C18—C12—C11	118.30 (13)
C4—C3—H3	119.6	C12—C13—C14	131.81 (14)
C2—C3—H3	119.6	C12—C13—H13	114.1
C3—C4—C5	119.70 (18)	C14—C13—H13	114.1
C3—C4—H4	120.1	C15—C14—C13	123.10 (14)
C5—C4—H4	120.1	C15—C14—S1	109.86 (12)
N1—C5—C4	121.90 (19)	C13—C14—S1	127.04 (12)
N1—C5—C6	116.64 (17)	C14—C15—C16	113.27 (15)
C4—C5—C6	121.46 (18)	C14—C15—H15	123.4
C5—C6—H6A	109.5	C16—C15—H15	123.4
C5—C6—H6B	109.5	C17—C16—C15	112.75 (16)
H6A—C6—H6B	109.5	C17—C16—H16	123.6
C5—C6—H6C	109.5	C15—C16—H16	123.6
H6A—C6—H6C	109.5	C16—C17—S1	112.05 (14)
H6B—C6—H6C	109.5	C16—C17—H17	124.0
C8—C7—C2	120.25 (16)	S1—C17—H17	124.0
C8—C7—H7	119.9	O3—C18—O2	122.61 (16)
C2—C7—H7	119.9	O3—C18—C12	124.76 (15)
C7—C8—C9	121.07 (18)	O2—C18—C12	112.62 (13)
C7—C8—H8	119.5	O2—C19—H19A	109.5
C9—C8—H8	119.5	O2—C19—H19B	109.5
C10—C9—C8	120.07 (17)	H19A—C19—H19B	109.5
C10—C9—H9	120.0	O2—C19—H19C	109.5
C8—C9—H9	120.0	H19A—C19—H19C	109.5
O1—C10—C9	125.42 (14)	H19B—C19—H19C	109.5
O1—C10—C1	114.06 (14)	C5—N1—C1	118.84 (14)
C9—C10—C1	120.52 (14)	C10—O1—C11	116.56 (12)
O1—C11—C12	107.82 (12)	C18—O2—C19	115.72 (14)
O1—C11—H11A	110.1	C17—S1—C14	92.07 (8)
			
N1—C1—C2—C7	−179.55 (15)	C12—C13—C14—C15	173.31 (18)
C10—C1—C2—C7	−0.1 (2)	C12—C13—C14—S1	−5.6 (3)
N1—C1—C2—C3	0.7 (2)	C13—C14—C15—C16	−178.11 (17)
C10—C1—C2—C3	−179.79 (14)	S1—C14—C15—C16	1.0 (2)
C7—C2—C3—C4	179.71 (18)	C14—C15—C16—C17	−0.8 (3)
C1—C2—C3—C4	−0.6 (3)	C15—C16—C17—S1	0.2 (3)
C2—C3—C4—C5	0.0 (3)	C13—C12—C18—O3	11.2 (3)
C3—C4—C5—N1	0.6 (3)	C11—C12—C18—O3	−168.58 (18)
C3—C4—C5—C6	−179.88 (19)	C13—C12—C18—O2	−169.61 (15)
C3—C2—C7—C8	179.74 (17)	C11—C12—C18—O2	10.6 (2)
C1—C2—C7—C8	0.1 (3)	C4—C5—N1—C1	−0.4 (2)
C2—C7—C8—C9	−0.2 (3)	C6—C5—N1—C1	179.99 (15)
C7—C8—C9—C10	0.3 (3)	C2—C1—N1—C5	−0.2 (2)
C8—C9—C10—O1	179.81 (15)	C10—C1—N1—C5	−179.71 (13)
C8—C9—C10—C1	−0.3 (2)	C9—C10—O1—C11	−1.8 (2)
N1—C1—C10—O1	−0.41 (19)	C1—C10—O1—C11	178.33 (12)
C2—C1—C10—O1	−179.90 (13)	C12—C11—O1—C10	175.71 (12)
N1—C1—C10—C9	179.70 (14)	O3—C18—O2—C19	0.0 (3)
C2—C1—C10—C9	0.2 (2)	C12—C18—O2—C19	−179.19 (17)
O1—C11—C12—C13	83.08 (19)	C16—C17—S1—C14	0.28 (18)
O1—C11—C12—C18	−97.16 (16)	C15—C14—S1—C17	−0.72 (15)
C18—C12—C13—C14	−178.52 (16)	C13—C14—S1—C17	178.33 (16)
C11—C12—C13—C14	1.2 (3)		

### Hydrogen-bond geometry (Å, º)

*Cg*3 is the centroid of the C1/C2/C7–C10 ring.

**Table:**

*D*—H···*A*	*D*—H	H···*A*	*D*···*A*	*D*—H···*A*
C19—H19*B*···*Cg*3^i^	0.96	2.89	3.505 (2)	123
C17—H17···O3^ii^	0.93	2.48	3.056 (2)	120

Symmetry codes: (i) *x*, *y*+1, *z*; (ii) *x*, *y*−1, *z*.
